# Harnessing Machine Learning, a Subset of Artificial Intelligence, for Early Detection and Diagnosis of Type 1 Diabetes: A Systematic Review

**DOI:** 10.3390/ijms26093935

**Published:** 2025-04-22

**Authors:** Rahul Mittal, Matthew B. Weiss, Alexa Rendon, Shirin Shafazand, Joana R N Lemos, Khemraj Hirani

**Affiliations:** 1 Diabetes Research Institute, University of Miami Miller School of Medicine, Miami, FL 33136, USA; mweiss16@student.nymc.edu (M.B.W.); arendon@student.nymc.edu (A.R.); joanalemos@miami.edu (J.R.N.L.); 2 Division of Endocrinology, Diabetes, and Metabolism, Department of Medicine, University of Miami Miller School of Medicine, Miami, FL 33136, USA; 3 School of Medicine, New York Medical College, Valhalla, NY 10595, USA; 4 Division of Pulmonary, Critical Care, and Sleep Medicine, Department of Medicine, University of Miami Miller School of Medicine, Miami, FL 33136, USA; sshafazand@med.miami.edu

**Keywords:** type 1 diabetes, machine learning, early detection, evidence synthesis, predictive modeling

## Abstract

Type 1 diabetes (T1D) is an autoimmune condition characterized by the destruction of insulin-producing pancreatic beta cells, leading to lifelong insulin dependence and significant complications. Early detection of T1D is essential to delay disease onset and improve outcomes. Recent advancements in artificial intelligence (AI) and machine learning (ML) have provided powerful tools for predicting and diagnosing T1D. This systematic review evaluates the current landscape of AI/ML-based approaches for early T1D detection. A comprehensive search across PubMed, EMBASE, Science Direct, and Scopus identified 1447 studies, of which 10 met the inclusion criteria for narrative synthesis after screening and full-text review. The studies utilized diverse ML models, including logistic regression, support vector machines, random forests, and artificial neural networks. The datasets encompassed clinical parameters, genetic risk markers, continuous glucose monitoring (CGM) data, and proteomic and metabolomic biomarkers. The included studies involved a total of 49,172 participants and employed case–control, retrospective cohort, and prospective cohort designs. Models integrating multimodal data achieved the highest predictive accuracy, with area under the curve (AUC) values reaching up to 0.993 in sex-specific models. CGM data and plasma biomarkers, such as CXCL10 and IL-1RA, also emerged as valuable tools for identifying at-risk individuals. While the results highlight the potential of AI/ML in revolutionizing T1D risk stratification and diagnosis, challenges remain. Data heterogeneity and limited model generalizability present barriers to widespread implementation. Future research should prioritize the development of universal frameworks and real-world validation to enhance the reliability and clinical integration of these tools. Ultimately, AI/ML technologies hold transformative potential for clinical practice by enabling earlier diagnosis, guiding targeted interventions, and improving long-term patient outcomes. These advancements could support clinicians in making more informed, timely decisions, thus reducing diagnostic delays and paving the way for personalized prevention strategies in both pediatric and adult populations.

## 1. Introduction

Type 1 diabetes (T1D) is a chronic autoimmune disorder characterized by the targeted destruction of insulin-producing pancreatic β-cells, resulting in insulin deficiency and the requirement for lifelong exogenous insulin therapy to regulate blood glucose levels [1]. In 2021, an estimated 8.4 million individuals globally were living with T1D, with projections ranging from 13.5 to 17.4 million by 2040 [2]. The economic burden of T1D in the United States alone is substantial, with lifetime management costs estimated at USD 813 billion per person, factoring in both direct healthcare expenditures and lost productivity. Despite progress in therapeutic strategies, delayed diagnosis remains a challenge, often resulting in severe complications such as diabetic ketoacidosis (DKA) [3,4,5]. Persistent hyperglycemia from late detection can further accelerate chronic complications, including retinopathy, nephropathy, neuropathy, and hearing loss [6,7,8,9,10,11,12]. In pediatric patients, such delays can significantly affect growth and long-term health outcomes [13].

Early detection through structured screening has demonstrated a reduced risk of DKA and better preservation of β-cell function, leading to improved metabolic control and reduced insulin requirements [14,15,16]. Screening programs based on islet autoantibody detection allow for risk stratification and early intervention, which can mitigate disease progression and improve long-term outcomes [14,17,18]. Incorporating such screening into routine pediatric care may reduce both acute and chronic disease burden [19,20].

The preclinical stage of T1D presents a unique opportunity for early detection, as it often involves measurable immunological, genetic, and metabolic changes that precede symptomatic disease [14,17]. However, traditional diagnostic tools often lack sensitivity and specificity, especially in heterogeneous populations. Most current methods detect the disease only after significant β-cell destruction, underscoring the urgent need for more advanced and predictive technologies.

In recent years, digital health technologies have advanced substantially, offering new tools that support both disease management and the potential for earlier detection. Systems integrating continuous glucose monitoring (CGM) with smart insulin delivery mechanisms have demonstrated efficacy in enhancing glycemic control through real-time glucose surveillance, automated insulin dose recording, and individualized therapeutic adjustments [21,22,23,24,25]. The advent of hybrid closed-loop systems, which partially automate insulin delivery in response to CGM data, has further reduced glycemic variability and eased the daily burden of disease management [26,27,28]. These integrated platforms not only enhance adherence and reduce the risk of hypoglycemia but also generate rich, continuous datasets. A recent real-world cohort study further substantiated the clinical utility of integrated CGM and smart insulin delivery systems [29]. The investigation assessed the impact of transitioning from conventional multiple daily injection (MDI) therapy to a smart MDI regimen utilizing the InPen™ smart insulin pen in conjunction with the Simplera™ CGM sensor. Over a 90-day follow-up period, the integrated system was associated with statistically significant improvements in glycemic metrics, including a reduction in mean sensor glucose concentrations and an increase in time in range (TIR), thereby reinforcing the effectiveness of such digital platforms in optimizing glycemic control under routine clinical conditions [29].

Artificial intelligence (AI), particularly machine learning (ML) algorithms, is emerging as a transformative tool in healthcare, offering unprecedented capabilities in disease prediction, diagnosis, and personalized management [30,31,32,33,34,35,36,37,38]. In this manuscript, we refer to ML, a subset of AI, as the primary computational methodology applied in the reviewed studies. While AI encompasses a broader range of technologies, including expert systems and natural language processing, our analysis focuses specifically on supervised and unsupervised ML models used for predictive modeling in T1D. These technologies excel in their ability to process and analyze large, multidimensional datasets, uncovering patterns and relationships that may not be detectable by traditional statistical techniques [39,40,41,42,43]. In the context of disease prediction and diagnosis, ML algorithms, such as logistic regression (LR), support vector machines (SVMs), random forests (RFs), and neural networks (NNs), have proved very useful [44,45,46,47]. These algorithms can be tailored to specific clinical challenges, ranging from early detection of diseases to optimizing therapeutic interventions. By learning from existing data, ML systems can identify subtle signals and complex interactions that may indicate an individual’s risk of developing a disease long before clinical symptoms manifest.

In the realm of T1D, ML-based approaches have shown significant potential as a tool to transform current paradigms of detection and management. T1D is characterized by a complex interplay of genetic, immunological, metabolic, and environmental factors [48,49,50,51,52,53,54,55], creating a wealth of data that traditional analytic methods may not be able to completely utilize. ML offers a means to integrate these diverse data streams, synthesizing them into predictive models capable of identifying individuals at risk for T1D with greater accuracy and earlier in the disease course. Neural networks can uncover nonlinear relationships within complex datasets [56,57,58], making them well-suited for exploring the multifactorial nature of T1D. Random forests and ensemble methods can provide robust predictions while offering interpretability [59,60,61,62,63], a critical feature in clinical decision-making. Additionally, SVMs can be used in T1D studies for their ability to separate complex data into well-defined categories, such as high-risk versus low-risk populations [64]. By leveraging ML algorithms, researchers and clinicians can not only improve the precision and timing of T1D diagnosis but also identify novel biomarkers and pathways that may serve as targets for preventive interventions. These technologies pave the way for a shift from reactive to proactive healthcare, where individuals at risk for T1D can be identified and monitored long before they experience symptomatic disease.

The primary objectives of this study were to systematically review and evaluate current applications of ML models, as a subset of AI, in the early detection and diagnosis of T1D. This includes assessing the types of ML algorithms used, their integration with clinical, genetic, metabolic, and CGM data, and their performance in predicting disease onset. The secondary objectives were to identify key challenges such as data heterogeneity, limited model generalizability, and barriers to clinical translation, as well as to highlight gaps in the literature and propose directions for future research. By providing a strategic framework for refining AI/ML-driven screening methodologies, this review aims to facilitate earlier interventions and improve clinical outcomes in T1D management.

## 2. Materials and Methods

### 2.1. Aims and Research Questions

This systematic review was designed to examine the use of ML approaches for the early detection and diagnosis of T1D. The aim was to assess how ML has been applied across clinical and research contexts, identify the most commonly used algorithmic models, and evaluate their performance based on key predictive metrics such as accuracy, sensitivity, specificity, and AUC. This review also explored the types of input data utilized for model development, including clinical parameters, genetic markers, metabolic profiles, CGM data, and multiomic biomarkers, with the goal of determining which data sources contribute most effectively to early disease prediction. Central to this review was an investigation into how these models are integrated into diagnostic workflows, their applicability in real-world settings, and the challenges encountered in terms of data heterogeneity, model generalizability, and clinical adoption. To structure the synthesis, this review focused on identifying which ML models have been employed for early-stage T1D prediction, the predictive value of various data types, the overall performance of these models, and the key limitations reported across studies. These aims provided a foundation for evaluating the current capabilities and future potential of ML in transforming early diagnosis and intervention strategies for T1D.

### 2.2. Search Strategy and Selection Criteria

This study was conducted according to the Preferred Reporting Items for Systematic Reviews and Meta-Analysis (PRISMA) guidelines [65]. A PRISMA checklist is provided as Supplementary Materials to ensure comprehensive reporting. This systematic review was designed *a priori* and registered in the PROSPERO database prior to the commencement of this study (registration number: CRD42024592995). A comprehensive search strategy was developed to identify studies focusing on the application of AI/ML for the early detection and diagnosis of T1D. The search was performed in October 2024 across four major electronic databases: PubMed, EMBASE, Science Direct, and Scopus. The search terms included combinations of relevant keywords: “type 1 diabetes” OR “T1D”, “artificial intelligence” OR “machine learning”, “predict” OR “detect”, “early intervention”, “predictive models”, “biomarkers”, and “precision medicine”. To ensure a broad retrieval of eligible studies, no publication date restrictions or search filters were applied, and the bibliographies of the included articles were manually reviewed for additional references.

Studies were included based on the following criteria: (1) original research articles utilizing AI or ML algorithms, such as LR, SVM, RF, and artificial neural networks (ANNs), to predict or detect early T1D onset; (2) clinical studies reporting predictive models, risk stratification, and performance metrics (such as accuracy, sensitivity, specificity, and area under the curve–receiver operating characteristic (AUC-ROC); (3) studies involving human subjects and published in peer-reviewed journals; and (4) articles written in English (Table 1). The exclusion criteria included studies focusing on type 2 diabetes (T2D) or non-T1D diagnoses, non-predictive research, animal or cadaveric studies, case reports, review articles, opinion pieces, conference abstracts, and pre-print manuscripts (Table 1).

The study selection process was conducted in three stages: title screening, abstract screening, and full-text review. Two independent reviewers (MBW and AR) systematically screened all identified records to ensure they met the inclusion criteria. Cohen’s κ was utilized to evaluate inter-rater agreement at each phase of the screening process [66]. The author agreement was assessed based on kappa values, with reference values of less than zero considered no agreement; 0.00–0.20 considered slight agreement; 0.21–0.40 considered fair agreement; 0.41–0.60 considered moderate agreement; 0.61–0.80 considered substantial agreement; and 0.81–1.000 considered almost perfect agreement. Based on previous studies, a κ value exceeding 0.6 was deemed indicative of substantial agreement, ensuring reliability for progression to subsequent steps [67,68,69,70]. In cases of disagreement between the two reviewers (MBW and AR), the senior authors (RM and KH) were consulted to reach a consensus.

All search results were imported into EndNote 21 (Clarivate Analytics, 1500 Spring Garden Philadelphia, PA 19130, USA) for reference management and deduplication. An initial automated process was employed using EndNote’s built-in duplicate detection algorithm, which identifies redundant records by comparing key bibliographic fields, including author names, article titles, journal names, and digital object identifiers (DOIs). To ensure maximum precision, a secondary manual curation was conducted to identify and resolve residual duplicates arising from inconsistencies in metadata formatting across databases. This two-tiered deduplication approach minimized data redundancy and ensured the integrity of the reference library prior to screening. The finalized set of unique records was subsequently exported for independent title and abstract screening in accordance with the PRISMA guidelines.

### 2.3. Assessment of Methodological Quality and Risk of Bias

The methodological quality of clinical evidence and risk of bias were assessed using the Joanna Briggs Institute (JBI) Critical Appraisal Tools for Diagnostic Test Accuracy [71]. This tool assesses key domains, including patient selection, interpretation of index test and reference standard results, diagnostic thresholds, timing between tests, and inclusion of all patients in the analysis [71]. Each domain was evaluated as “Yes”, “unclear”, “No”, or “Not Applicable”, and a resulting overall score was given. Two authors (AR and MBW) assessed each study and consulted to achieve consensus. Any disagreements were resolved by consulting the senior authors (RM and KH). The quality assessment for each included study was summarized and reported.

### 2.4. Data Extraction and Analysis

Data extraction was performed using a standardized form, capturing study design, sample size, patient demographics, data integration methods (such as proteomics, metabolomics, genomics, and continuous glucose monitoring [CGM]), ML models employed, and performance outcomes, including feature selection strategies. The data from each study were independently extracted and evaluated by two independent reviewers.

## 3. Results

**Figure 1:**
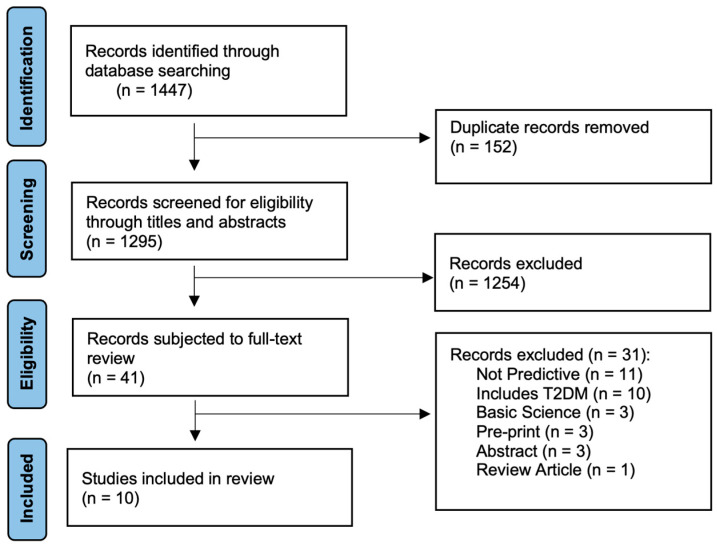
PRISMA diagram showing the study selection process.

This systematic search yielded a total of 1447 studies from the selected databases (Figure 1). Following the initial search, duplicate records were identified and removed, resulting in a total of 1295 unique studies available for further screening. The titles and abstracts of these 1295 studies were then carefully reviewed to evaluate their relevance to the research question. After this initial screening phase, 41 studies were deemed potentially eligible and were retrieved for a more detailed full-text review. During the full-text review, each study was evaluated against the predefined inclusion and exclusion criteria to ensure that only those meeting the specific parameters of this systematic review were included. This rigorous evaluation process resulted in the final selection of 10 studies that met all the necessary criteria and formed the foundation for the analysis and synthesis of the findings presented in this systematic review (Figure 1).

We calculated Cohen’s κ score to assess inter-rater agreement during each screening phase. The results indicated substantial agreement, with a score of 0.739 (95% CI: 0.643, 0.834) for phase 1 (title and abstract screening) and 0.715 (95% CI: 0.481, 0.950) for phase 2 (full-text screening).

### 3.1. Study Characteristics and Outcomes

The reviewed studies, comprising a total of 49,712 participants, included diverse study designs such as case–control, retrospective, and prospective cohort studies (Table 2). The participants ranged from children under 15 years to adults, including individuals at genetic or familial risk for T1D. All the studies focused on T1D as the primary exposure, comparing outcomes against non-diabetic controls. The studies identified predictive markers, including trace elements [72], proteomic and metabolomic profiles [73,74,75], T1D-associated autoantibodies [72,76], and demographic or clinical features. The findings demonstrated classification accuracies of up to 92.5%, enabling early prediction of T1D development and improving diagnostic timing. These results emphasize the potential of integrating biomarkers and advanced analytics for early detection and effective management of T1D.

### 3.2. Summary of the Quality Assessment

**Figure 2:**
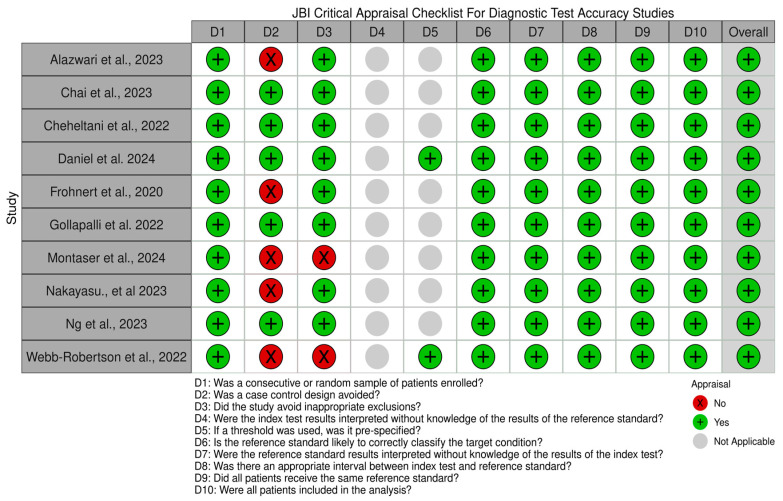
**Risk of bias assessment of the included studies.** This figure presents the risk of bias assessment of the included studies [12,72,73,74,75,76,77,78,79,80] based on the Joanna Briggs Institute (JBI) Critical Appraisal Tools. The color coding represents the level of bias: green denotes low risk of bias, yellow indicates unclear risk, and red suggests high risk of bias.

The methodological quality of the included studies, evaluated using the JBI Critical Appraisal Checklist for Diagnostic Test Accuracy, is summarized in Figure 2. Most of the studies met key quality criteria, such as appropriate patient sampling and unbiased interpretation of reference standards. However, some lacked pre-specified diagnostic thresholds, leading to variability in reporting quality.

### 3.3. Comparative Performance of ML Models

**Figure 3:**
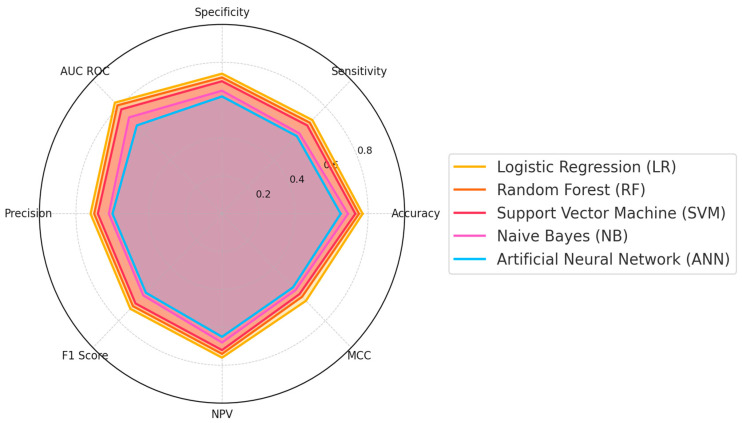
**Comparison of AI model performance across multiple metrics.** This radar plot compares the performance of five AI models—logistic regression (LR), random forest (RF), support vector machine (SVM), Naïve Bayes (NB), and artificial neural network (ANN)—based on eight key metrics: accuracy, sensitivity, specificity, AUC ROC, precision, F1 Score, Negative Predictive Value (NPV), and Matthews Correlation Coefficient (MCC). Each axis represents one metric, with higher values indicating better performance. The filled areas for each model illustrate their strengths and weaknesses across these metrics, highlighting the superior overall performance of the logistic regression and random forest models compared to the others. The plot emphasizes the balanced evaluation of AI models in predicting type 1 diabetes.

The comparative performance of five ML models was determined from the included studies in this review, namely, RF, LR, SVM, Naïve Bayes (NB), and artificial neural network (ANN). These models were evaluated across eight key metrics: accuracy, sensitivity, specificity, precision, F1 Score, Negative Predictive Value (NPV), Matthews Correlation Coefficient (MCC), and AUC-ROC. These metrics provided a comprehensive assessment of each model’s predictive capabilities for T1D. As shown in the radar plot in Figure 3, RF and LR demonstrated the strongest overall performance, performing well across most metrics, including accuracy and AUC-ROC. SVM performed well on specific metrics, such as sensitivity and precision, indicating its reliability in certain classification tasks. NB showed moderate and consistent performance, achieving reasonable values for NPV and specificity. In contrast, ANN’s performance was relatively lower, as suggested by its proximity to the center across most metrics. The performance characteristics and conclusions of the studies utilizing these ML models for T1D prediction are discussed in detail in the following sections. A meta-analysis was not feasible owing to the heterogeneity in the outcomes reported in the included studies.

### 3.4. ML for Predicting T1D in Children

Alazwari et al. performed ML analyses on a dataset of almost 1200 individuals 0–14 years old, including multiple versions of LR, RF, SVM, NB, and ANN [12]. The full LR was the best-performing predictive model, with an accuracy of 0.77 (CI: 0.7116, 0.8264), a precision of 0.7, and an AUC ROC of 0.83. The second best was the full RF model with an accuracy of 0.75 (CI: 0.7019, 0.8181), a precision of 0.64, and an AUC ROC of 0.81. The model with the lowest performance was the reduced ANN with two hidden layers, with statistics of 0.65 (CI: 0.5877, 0.7172), 0.5, and 0.66, respectively. Their study also evaluated key performance indicators (KPIs) for T1D diagnosis based on socio-demographic, potential genetic, and environmental factors, and they found that significant predictors include early exposure to cow’s milk (OR = 2.92, *p* < 0.001), birth weight > 4 kg (OR = 3.11, *p* = 0.007), rural residency (OR = 3.74, *p* < 0.001), family history of diabetes, and maternal age > 25 years [12].

### 3.5. Clinical and Trace Elements as Predictors of T1D Risk

Chai et al. analyzed a large number of clinical and trace element data to determine the variables with the highest predictive value for T1D by comparing 105 T1D patients with 105 matched non-T1D patients, modeling male and female participants separately [72]. It was determined that the predictive model for males should include serum triglyceride, total protein, and serum magnesium, whereas for females, it should include apolipoprotein A, creatinine, total iron, selenium (Se), and the zinc (Zn)-to-copper (Zn/Cu) ratio. Subsequently, an LR was conducted based upon these findings, with “Model A” combining clinical and trace elements, resulting in an AUC ROC in the male cohort of 0.993 and in the female cohort of 0.951, as well as scores of 0.882 and 0.945 in the validation sets with clinical significance. Models were also conducted for clinical parameters and trace elements alone, the so-called Model B and Model C, respectively, which performed at slightly lower levels when compared to Model A [72].

### 3.6. Identifying Misdiagnosed Adult-Onset T1D

Cheheltani et al. developed an ML model to identify cases of adult-onset T1D misdiagnosed as T2D [77]. Using retrospective data from Ambulatory Electronic Medical Records (AEMRs), the algorithm highlighted age, BMI/weight, therapy history, and HbA1c/blood glucose values as top predictors of misdiagnosis. A model for T1D was conducted, which resulted in an AUC of 0.81. At a low recall level of 10%, the model achieved a precision of 17%, markedly higher than the <1% incidence rate of misdiagnosis at the time of initial T2D diagnosis [77].

### 3.7. Early Detection of T1D Using Data from Electronic Health Records

Daniel et al. utilized a dataset of 952,402 children from electronic health records from the Welsh Brecon dataset to create an ML algorithm [78]. The algorithm used 26 predictors covering demographics, such as age and sex, and clinical predictors, such as polyuria, headache, and antibiotic usage, among others. The model identified 71.6% (95% CI: 68.8–74.4) of T1D cases within 90 days prior to diagnosis when set to trigger alerts in 10% of the cases. Diagnosis was anticipated, on average, by 9.34 (95% CI: 7.77–10.9) days. When set to trigger alerts in 5% and 3.1%, they displayed sensitivities of 64.2% (95% CI: 61.2–67.2) and 59.8% (95% CI: 56.7–62.9), respectively [78].

### 3.8. Multi-Omic Biomarkers in T1D Progression

Frohnert et al. used an integrative ML approach to identify predictors of islet autoimmunity (IA) and progression to T1D in a high-risk pediatric cohort [75]. The study involved 67 children evaluated at four time points, measuring genetic, immunologic, metabolomic, and proteomic biomarkers. Their Repeated Optimization for Feature Interpretation (ROFI) model, recursive feature elimination model, and combination model attained an AUC of 0.92, 0.82, and 0.64, respectively, for progression to T1D. The key predictors of IA included changes in serum ascorbate, 3-methyl-oxobutyrate, and the PTPN22 polymorphism, while serum glucose, ADP fibrinogen, and mannose were significant for diabetes progression [75].

### 3.9. Stacking Ensemble Models for Diabetes Detection

Gollapalli et al. proposed a stacking ensemble model for detecting pre-diabetes, T1D, and T2D using a Saudi Arabian dataset [79]. The model combined multiple classifiers to enhance performance. It was evaluated using metrics such as accuracy, sensitivity, specificity, and AUC ROC. Their study also evaluated a dataset comprising clinical and demographic features, including five principal risk factors that were identified from the Stacking model, including education, diabetic status, insulin, nutrition, and sex. The researchers focused on these factors to allow for robust feature selection and model training in the computation (e.g., education, nutrition, insulin use, and sex). The model achieved the highest accuracy for T2D detection (97.3%) compared to T1D (95.1%) and pre-diabetes (92.6%). Sensitivity values were 96% for T2D, 93% for T1D, and 90% for pre-diabetes. The high level of sensitivity shows strong true positive rates across groups, with T2DM showing the most robust detection. Specificity followed a similar trend, with 98% for T2D, 94% for T1D, and 91% for pre-diabetes, reflecting precise differentiation of non-diabetic cases. The AUC scores were highest for T2D (0.98) compared to T1D (0.96) and pre-diabetes (0.91), emphasizing the model’s exceptional performance in distinguishing T2D. These findings highlight the model’s superior efficacy in detecting T2D, with slightly reduced but still strong performance for T1D and pre-diabetes. One limitation of the study is that it may not be generalized outside of the Saudi Arabian population without taking into consideration environmental and lifestyle differences across global regions and variations in the obesity epidemic by country. The dataset included clinical and demographic variables, emphasizing the integration of diverse features for training and testing. These results underscore the stacking ensemble model’s effectiveness in diabetes detection, offering a promising tool for early diagnosis and intervention [79].

### 3.10. CGM Data and ML for Early T1D Prediction

Montaser et al. performed an exploratory study leveraging one-week CGM data and ML to predict the risk of developing T1D [76]. The researchers stratified participants by high- vs. low-risk groups based on CGM data. Forty-two healthy relatives of people with T1D with a mean ± SD age of 23.8 ± 10.5 years, HbA1c (glycated hemoglobin) of 5.3% ± 0.3%, and BMI (body mass index) of 23.2 ± 5.2 kg/m^2^ with zero (low-risk; N = 21) and ≥2 (high-risk; N = 21) antibodies were enrolled in an NIH (National Institutes of Health)-funded TrialNet ancillary study. The exploratory study utilized CGM data to train and test ML models for classification, with an emphasis on early detection. The ML-enhanced classification models demonstrated strong predictive performance, with the best-performing model achieving an AUC of 0.92, showing discrimination between the two groups. The ML models demonstrated a significant ability to distinguish between these groups, with the high-risk group showing markedly higher glucose variability (mean standard deviation of glucose of 34 mg/dL in the high-risk group vs. 22 mg/dL in the low-risk group, *p* < 0.001). The model achieved a sensitivity of 89%, reflecting its ability to correctly identify individuals at risk, and a specificity of 87%, ensuring a high level of identification of those not at risk. However, the sensitivity and specificity were still higher in the Gollapalli et al. models. Additionally, metrics such as time-in-range (TIR) showed significant differences, with the high-risk group spending 15% less time in the optimal glucose range compared to the low-risk group (*p* < 0.01). These comparisons underline the effectiveness of CGM data coupled with ML techniques in identifying individuals at elevated risk of developing T1D. One limitation of the study is its dependence on continuous glucose monitoring, which may be resource- and labor-intensive. These results suggest that integrating at-home CGM data with advanced ML algorithms provides a promising approach to identify individuals at high versus low risk of T1D classification, enabling earlier self-monitoring [76].

### 3.11. Plasma Proteins as Predictors of T1D

The research conducted by Nakayasu et al. investigated plasma protein biomarkers as predictors of persistent autoantibodies and T1D development [73]. The researchers aimed to identify distinct differences between groups based on their risk profiles. The analysis revealed that individuals who developed persistent autoantibodies had significantly elevated levels of inflammatory and immune-regulatory proteins, such as CXCL10 (an inflammatory chemokine that binds to CXCR3 receptor), with a mean difference of 1.8-fold (*p* < 0.001), and IL-1RA (mean difference: 2.2-fold, *p* < 0.01), compared to those who remained autoantibody-negative. The CXCL10 mean concentration was 105 pg/mL vs. 58 pg/mL (*p* < 0.001), and the IL-1RA mean concentration was 182 pg/mL vs. 82 pg/mL (*p* < 0.01). Furthermore, six months prior to the onset of autoimmunity, the high-risk group exhibited a notable increase in markers related to immune activation and beta-cell stress. This stress can lead to apoptosis of beta-cells prior to diagnosis, which may lead to further AI investigation on the timing of T1D diagnosis relative to these inflammatory markers. The predictive model incorporating these biomarkers achieved an AUC of 0.89, with a sensitivity of 85% and specificity of 82%, significantly outperforming traditional risk assessments. Comparisons between the high-risk and low-risk groups demonstrated distinct proteomic signatures, suggesting the utility of plasma biomarkers for early intervention strategies in individuals predisposed to T1D. Overall, although focusing on biomarkers provides specific insights into immune dysregulation preceding autoimmunity, it may also limit the broader applicability in the field of AI by not taking into consideration genetic, environmental, and lifestyle factor contributions [73]. 

### 3.12. Islet Autoantibody Levels as Predictors of T1D

A prospective cohort study by Ng et al. aimed to evaluate the utility of islet autoantibody (IAb) levels in predicting T1D in autoantibody-positive children [80]. Using data from prospective cohort studies in Finland, Germany, Sweden, and the USA, the researchers analyzed 1403 children who developed islet autoantibodies, out of which 523 progressed to diabetes. The study investigated how IAb levels could enhance predictive power compared to qualitative IAb positivity indicators. Data were collected from 24,662 children at genetic or familial risk of developing islet autoimmunity and diabetes. The following autoantibodies were measured: insulin autoantibodies (IAAs), glutamic acid decarboxylase autoantibodies (GADAs), and insulinoma-associated antigen-2 autoantibodies (IA-2As). Diabetes prediction models were developed using multivariate logistic regression with inverse probability censored weighting (IPCW). These models were trained and validated using 10-fold cross-validation, and the concordance index (C index) was used to measure predictive power. The researchers demonstrated that an ML model with only demographic and genetic covariates (sex, family history, HLA risk group, and age at seroconversion) achieved a C index of 0.61 (95% CI: 0.58, 0.63) for a 10-year follow-up. The impact of adding IAb positivity indicators through the inclusion of IAA, GADA, and IA-2A positivity improved the C index to 0.72 (95% CI: 0.71, 0.74). Additionally, using IAb levels alone (without baseline covariates) maintained a C index of 0.76 (95% CI: 0.75, 0.76). Applying the predictive model to follow-up periods showed the best performance for shorter follow-up durations, with a C index of 0.82 (95% CI: 0.81, 0.83) for 2 years. Even in an 11-year follow-up time, the performance of the model remained reasonable for longer durations, maintaining a C index of 0.76 (95% CI: 0.75, 0.76) even at 11 years. A third IAb test, approximately 1.5 years after seroconversion, further improved the prediction accuracy, yielding a C index of 0.78 (95% CI: 0.77, 0.78) for a 10-year follow-up. Although the primary statistical model used was a logistic regression model with IPCW, the study also utilized survival analysis to understand disease progression. The Cox proportional hazards model was likely used to assess the time-to-event (diabetes diagnosis) data, evaluate the hazard ratios (HRs) for different IAb levels and their predictive value, and estimate how different autoantibody levels influence the rate of diabetes onset over time. Thus, the study contributes to refining screening strategies for T1D and can facilitate the selection of participants for preventive interventions.

### 3.13. Multi-Modal AI for T1D Prediction

A study by Webb-Robertson et al. aimed to evaluate whether a combination of genetic, immunologic, and metabolic factors measured during infancy could predict the onset of T1D by age 6 years [74]. The study used data from The Environmental Determinants of Diabetes in the Young (TEDDY) cohort, including children from Finland, Germany, Sweden, and the United States. A total of 702 children with complete data from TEDDY were analyzed, of whom 11.4% developed T1D by age 6. The data collected included genetic risk scores (GRSs), human leukocyte antigen (HLA) genotyping, islet autoantibody (IAAb) status for insulin autoantibody (IAA), glutamic acid decarboxylase autoantibody (GADA), and insulinoma-associated antigen-2 autoantibody (IA-2A), as well as metabolomics data from blood samples at ages 3, 6, and 9 months. Infant attributes such as birth weight, diet, and family history were collected throughout the study period. Using ML analysis, a feature selection for the Naïve Bayes classifier with Repeated Optimization for Feature Interpretation (ROFI) was used to identify key predictors. The predictive model achieved an AUC of 0.84, indicating strong classification ability [74]. Regarding the optimal predictors, a model using only 3- and 9-month measurements had a similar AUC, suggesting early-life metabolic markers play a critical role. Some key metabolites identified included altered sugar metabolism (fructose, xylulose), purine degradation (uridine, inosine), and pentose phosphate pathway changes linked to future T1D development. Additionally, the importance of an early screening age showed that a single blood draw at 9 months provided the most predictive power, though adding a 3-month measurement improved accuracy slightly. The model correctly identified 38% of future T1D cases using a combination of IAAb, metabolomics, and genetic markers. Thus, early metabolic changes indicated a predisposition to T1D, and the strongest predictors of T1D were IAAb positivity, specific genetic markers (HLA and GRS), and key metabolic changes. This ML analysis encourages screening at 9 months of age as an optimal timeline for identifying at-risk children. These results demonstrate the synergistic value of integrating metabolite, genetic, and autoimmunity data for early and accurate T1D prediction in children [74].

## 4. Discussion

This systematic review evaluates the current landscape of ML models in the early detection and diagnosis of T1D by synthesizing findings from studies utilizing clinical, genetic, metabolic, proteomic, and environmental datasets. The findings highlight significant advancements in various ML models, demonstrating their substantial potential in improving predictive accuracy, with several achieving area AUC values exceeding 0.90 [74,75,76]. The ability of ML algorithms to integrate multimodal data sources, including genetic, metabolic, immunologic, and CGM parameters, has led to superior classification and risk stratification capabilities compared to traditional statistical models [12,72,73,74,75,76,77,78,79,80]. These advancements highlight the capacity of AI/ML tools to capture complex, nonlinear relationships within heterogeneous datasets, enhancing the early detection and diagnosis of T1D.

The studies reviewed reveal that LR, SVMs, RFs, and ANNs are among the most frequently employed ML models in T1D prediction [12,79]. The choice of algorithm varies depending on the dataset structure, sample size, and specific predictive markers included. Logistic regression remains widely utilized due to its interpretability and robustness in handling structured clinical and demographic data [12]. Notably, ensemble methods, including stacking models that integrate multiple classifiers, have demonstrated enhanced predictive accuracy by leveraging the strengths of individual algorithms while mitigating their respective limitations [79].

The integration of CGM data into predictive modeling has yielded promising results, as continuous glucose fluctuations provide dynamic physiological insights that static biomarkers cannot capture [76]. ML models trained on CGM-derived features have demonstrated a remarkable ability to distinguish high-risk individuals from those with stable glucose regulation, often identifying pre-diabetic states well before clinical diagnosis [76]. By leveraging real-time glucose variability patterns, these models enhance early intervention efforts, facilitating timely therapeutic adjustments that may delay or even prevent T1D onset. Furthermore, studies incorporating plasma biomarkers, such as inflammatory cytokines and proteomic signatures, reinforce the biological plausibility of ML-driven predictions, as these molecular signals reflect immune dysregulation and beta-cell stress preceding disease manifestation [73,74].

Data extracted from EHRs further highlight the feasibility of AI/ML-assisted early diagnosis in clinical practice [78]. The retrospective analysis of large-scale patient registries has enabled the identification of subtle clinical indicators that precede T1D development, such as polyuria, recurrent infections, and metabolic dysregulation [78]. By integrating historical patient data with real-time physiological monitoring, ML-based approaches offer an unprecedented level of predictive precision that surpasses conventional screening methodologies [78]. Importantly, these models exhibit potential in identifying cases of adult-onset T1D misdiagnosed as type 2 diabetes, which has significant implications for optimizing treatment strategies and minimizing long-term complications [77].

The use of multiomic biomarkers has further refined predictive modeling by incorporating genetic predisposition, metabolomic signatures, and autoantibody profiles into risk assessment frameworks [74,75,80]. Studies analyzing metabolomic alterations during infancy have demonstrated that early-life metabolic shifts serve as strong indicators of future T1D progression, reinforcing the concept that disease onset is preceded by a prolonged preclinical phase characterized by molecular perturbations [67,68]. The combination of genetic risk scores with autoantibody measurements and metabolic markers has produced robust classifiers capable of identifying high-risk individuals with remarkable specificity [74,80]. Such integrative approaches enhance disease staging and improve patient stratification for clinical monitoring, providing a foundation for personalized risk assessment in genetically susceptible populations [74].

Despite the heterogeneity in datasets and study methodologies, a consistent trend across investigations is the superiority of ML-driven predictive models over traditional statistical techniques in capturing complex disease trajectories. The ability to extract meaningful insights from vast, multidimensional datasets has redefined the paradigm of early T1D detection, enabling a shift from symptomatic diagnosis to preemptive risk identification.

## 5. Limitations

While ML models for the early detection and diagnosis of T1D show promising results, there are challenges and limitations that need to be addressed. One significant limitation is the variability in datasets used for model development and validation. Studies often rely on specific cohorts or geographically restricted populations, such as the Saudi Arabian or European datasets, which may not generalize well to other ethnic or regional groups. This lack of diversity hinders the broader applicability of predictive algorithms across global populations. Moreover, the sample sizes in several studies are limited, especially when stratifying participants into subgroups such as high- and low-risk individuals. Small sample sizes can introduce bias, reduce statistical power, and lead to overfitting, compromising the reliability of the models.

Another critical issue involves data quality and accessibility. ML/AI models rely on accurate, high-resolution data from clinical, genetic, and environmental sources. However, inconsistencies in data collection, variations in biomarker measurements, and gaps in EHRs can negatively impact model performance. While CGM data are valuable, their availability may be limited in lower-resource settings due to the cost and accessibility of devices rather than the complexity of data collection itself. Similarly, genetic and proteomic data, though highly informative, may still be expensive and less readily available in large-scale screening efforts, particularly in resource-constrained environments.

The interpretability of ML models remains another challenge. ML algorithms, particularly deep learning-based approaches, often function as “black boxes”, offering limited transparency into how predictions are made. While some studies highlight key predictors, such as genetic risk markers or metabolic profiles, the underlying biological mechanisms and interactions remain incompletely understood. This lack of explainability poses challenges for clinical adoption, as healthcare providers require confidence in the rationale behind ML-generated predictions to integrate them into patient care effectively. Additionally, despite the high sensitivity and specificity reported in studies, model performance can vary significantly when tested on independent datasets or under real-world conditions.

## 6. Perspectives for Clinical Practice

The application of ML in the management of T1D offers a promising pathway to enhance clinical care through advanced analytics and individualized risk evaluation. Studies have shown that ML models can effectively identify individuals who are more likely to develop T1D, thereby supporting earlier and more precise intervention strategies. For instance, leveraging features from HER and analyzing CGM data through ML algorithms have both contributed to earlier recognition and improved diagnostic timing [76,78].

These insights hold significant potential for implementation across pediatric endocrinology, primary care, and early screening initiatives. ML-based tools can enrich clinical workflows by detecting subtle biological or behavioral indicators that precede clinical symptoms. This enables timely lifestyle adjustments or therapeutic measures. In addition, ML systems can be integrated into digital health technologies, such as smart insulin devices, automated insulin delivery systems, and mobile health applications, to assist with real-time clinical support [21,22,23,24,25].

To fully harness these advancements, upcoming initiatives should emphasize provider training, seamless integration with current electronic platforms, and inclusive access to the required technology. Through these efforts, ML can play a central role in advancing early-stage, personalized approaches to diabetes care.

## 7. Conclusions

Recent progress in AI/ML has substantially improved the identification of individuals at risk for T1D at an earlier stage compared to conventional diagnostic methods. This systematic review highlights the effectiveness of predictive models in integrating various data modalities, including genetic, metabolic, immunologic, and continuous glucose monitoring parameters, enabling more precise risk stratification. The expanding role of digital health technologies in enhancing these models further highlights their potential to support early intervention strategies aimed at delaying or preventing disease onset.

Incorporating real-time analytics and wearable biosensors into predictive frameworks presents a promising avenue for future research. Continuous glucose monitoring and other physiological biomarkers facilitate longitudinal tracking of at-risk individuals, enhancing early detection algorithms by enabling dynamic and personalized risk assessments. Further exploration of decentralized learning approaches, which support collaborative model training across institutions while maintaining data privacy, may improve algorithm performance and address ethical concerns related to security and confidentiality.

With continued advancements in computational methodologies, AI/ML have the potential to revolutionize the early detection of T1D. To maximize their clinical utility, it is imperative to address the existing limitations and rigorously validate predictive models in diverse, real-world settings. The implementation of these technologies has the potential to shift the diagnostic paradigm from late-stage disease identification to proactive risk stratification and early therapeutic intervention. This shift could enhance patient outcomes, improve disease management strategies, and reduce the long-term burden of T1D.

## Figures and Tables

**Table 1:** Inclusion and exclusion criteria.

Inclusion Criteria	Exclusion Criteria
Clinical studies using ML or AI for early detection/diagnosis of T1D	Studies lacking predictive modeling
Studies reporting risk stratification and model performance metrics (such as accuracy, sensitivity, specificity, AUC-ROC)	Included T2D or other non-T1D diagnoses
Published in a peer-reviewed journal	Review articles
Involving human subjects	Animal and cadaveric studies
Written in English	Case reports

ML, machine learning; AI, artificial intelligence; T1D, type 1 diabetes; T2D, type 2 diabetes.

**Table 2:** A summary of the included studies.

Reference	Study	Sample Size/Population	Comparison	Outcomes/Study Conclusions
Alazwari et al., 2023 [12]	Case–control study	A total of **1142** children <15 years with a confirmed diagnosis of T1D between 2010 and 2020	Non-diabetic controls	Significant KPIs included the following: - Early exposure to cow’s milk, birth weight > 4 kg. - Family history of T1D in first-degree relatives and siblings and second. - Maternal age (25–35 years) and greater than 35 years.
Chai et al., 2023 [72]	Retrospective cohort (EMRs)	A total of **105** T1D patients with negative insulin autoantibodies (zinc transporter8, anti-islet cell antibody, anti-glutamate decarboxylase antibody, anti-tyrosine phosphatase antibody, anti-insulin antibody, islet antigen-2 autoantibodies), 2019–2020	Non-diabetic controls	- Significantly elevated serum Fe, Cu, and Zn and decreased Mg were demonstrated in T1D males, and lower levels of trace elements were found in females. - The correlation between clinical parameters and trace elements was more obvious in females and liver function (LFTs) were more commonly found in correlation with trace elements. - The combination of Mg, TG, and TP in males and the combination of Fe, Se, the Zn/Cu ratio, Cre, and Apo A could be used as efficient parameters for auxiliary prediction in T1D patients with negative autoantibodies and provide reference alarm for individuals with high-risk of T1D morbidity.
Cheheltani et al., 2022 [77]	Retrospective cohort (AEMRs)	A total of **15,881** patients with type 1	Patients misdiagnosed as type 2 cohort	- Age, BMI/weight, therapy history, and HbA1c/blood glucose values among top predictors of misdiagnosis. - Model precision at low levels of recall (10%) was 17%, compared to a <1% incidence rate of misdiagnosis at the time of the first type 2 diabetes encounter in AEMR.
Daniel et al., 2024 [78]	Retrospective cohort (EHRs)	A total of 1829 children younger than 15 years with type 1 DM development	Non-diabetic controls	- Reduced number of days to diagnosis for children, on average, by an estimated 9.34 days (95% CI 7·77–10·9).
Frohnert et al., 2020 [75]	Case–control study	A total of 2547 children in the DAISY cohort at increased DM risk, first-degree relatives of patients with type 1 diabetes (FDRs), and general-population children with type 1 diabetes susceptibility HLA DR-DQ genotypes identified by newborn screening, recruited between 1993 and 2004	Non-diabetic control family vs. children with increased DM risk	- The ROFI-P3 algorithm can identify and evaluate known and novel predictors of development of IA and progression to diabetes across disparate data sources. - In children with high-risk HLA genotypes, changes in the relative abundance of certain proteins, such as high cystatin-F, FCRL3 (Fc receptor-like protein 3), KLRK1, MMP-2, and activin, were found. - Metabolites, such as higher glucose, mannose, and ribose, were predictive and elevated in children with future T1D diagnosis. - Genetic markers—SSRP1, a protein involved in DNA repair, and CSK21—predicted the development progression of diabetes. - Seroconversion was associated with an altered antioxidant profile.
Gollapalli et al., 2022 [79]	Retrospective cohort (EMRs)	A total of 2067 patients with cancer (n = 93), dementia (n = 152), and diabetes (n = 1822)	Non-diabetic controls	- Computational intelligence techniques were used to distinguish and predicted three types of diabetes, namely: ○ T1D; ○ T2D; ○ Pre-diabetes. - The proposed SVM model achieved the highest testing prediction classification accuracy of 92.5% as compared with the proposed KNN model. - After performing the permutation feature importance analysis, it appeared that education, AntiDiab, insulin, nutrition, and sex were the most important features affecting the model’s ability to predict significantly.
Montaser set al., 2024 [76]	Case–control study	A total of 56 individuals without a history of diabetes and fasting plasma glucose < 126 mg/dL classified as normoglycemia (n = 33) or pre-diabetes (n = 21)	Non-diabetic controls	- Individuals with glucose ranging from normoglycemic to pre-diabetes exhibited clear heterogeneity in four distinct physiologic processes that contribute to disordered glucose metabolism, including muscle IR, β-cell dysfunction, impaired incretin effect, and hepatic IR. - The majority of individuals exhibited a single dominant metabolic subphenotype or two codominant phenotypes.
Nakayasu et al., 2023 [73]	Case–control study (TEDDY study)	Untargeted proteomics of 2252 samples from 184 individuals identifying 376 regulated proteins	Non-diabetic controls	- AI identified and validated 83 biomarkers of IA and T1D development prior to the onset of the disease. - ML analysis identified panels of proteins that can predict both the development of persistent autoantibodies with normoglycemia and T1D even 6 months prior to the appearance of the autoimmune response.
Ng et al., 2023 [80]	Prospective cohort studies	A total of 24,662 children at increased genetic or familial risk of developing islet autoimmunity and diabetes	Non-diabetic controls	- Consideration of quantitative patterns of IAb levels improved the predictive power for type 1 diabetes in IAb-positive children beyond qualitative IAb positivity status.
Webb-Robertson et al., 2022 [74]	Case–control study (TEDDY study)	A total of 702 children with all data sources measured at ages 3, 6, and 9 months, 11.4% of whom progressed to T1D by age 6 years	Non-diabetic controls	- Biomarkers that can accurately predict risk of T1D in genetically predisposed children can facilitate interventions to delay or prevent the disease. - Machine-learning-based feature selection yielded classifiers based on disparate demographic, immunologic, genetic, and metabolite features. - Accuracy of the model using all available data evaluated by the area under a receiver operating characteristic curve was 0.84. - Reducing to only 3- and 9-month measurements did not reduce the area under the curve significantly. - Metabolomics had the largest value when evaluating the accuracy at a low false-positive rate. - The metabolite features identified as important for progression to T1D by age 6 years pointed to altered sugar metabolism in infancy. Integrating this information with classic risk factors improves prediction of the progression to T1D in early childhood.

Abbreviations: EMRs, electronic medical records; KPI, key performance indicators; AEMRs, Ambulatory Electronic Medical Records; EHRs, electronic health records; DAISY, Diabetes Autoimmunity Study in the Young; TEDDY, The Environmental Determinants of Diabetes in the Young.

## Data Availability

Data of PRISMA checklist [81] is available in Supplementary Materials.

## Supplementary Materials

The following supporting information can be downloaded at: https://www.mdpi.com/article/10.3390/ijms26093935/s1.
